# Change in bowel habit, abdominal pain, and a palpable abdominal mass in a 58‐year‐old female

**DOI:** 10.1002/ccr3.1171

**Published:** 2017-11-09

**Authors:** Julian Peacock, Zane Perkins

**Affiliations:** ^1^ Department of General Surgery The Royal London Hospital Whitechapel London E1 1BB UK

**Keywords:** Colorectal disease, diverticular disease, general surgery, giant colonic diverticulum

## Abstract

Giant colonic diverticulum is a rare complication of diverticulosis, which may present in the acute or chronic setting. Colonic resection and en bloc resection of the diverticulum are the favored management, however, conservative treatment remains an option that could be considered in asymptomatic patients with minimal symptoms.

## Introduction

**Figure 1:**
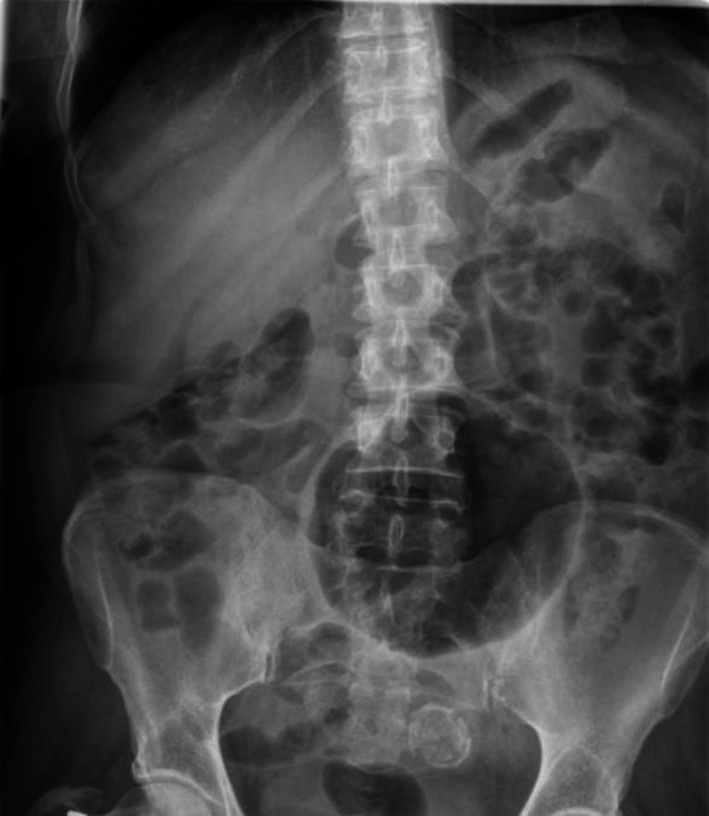
Abdominal radiograph.

**Figure 2:**
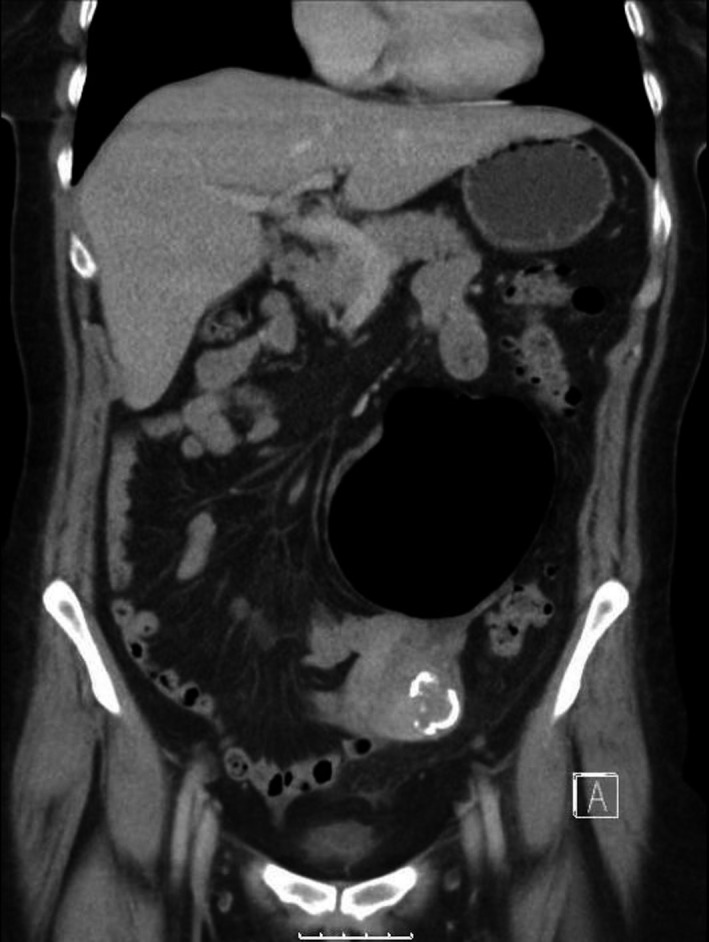
CT image (coronal view).

**Figure 3:**
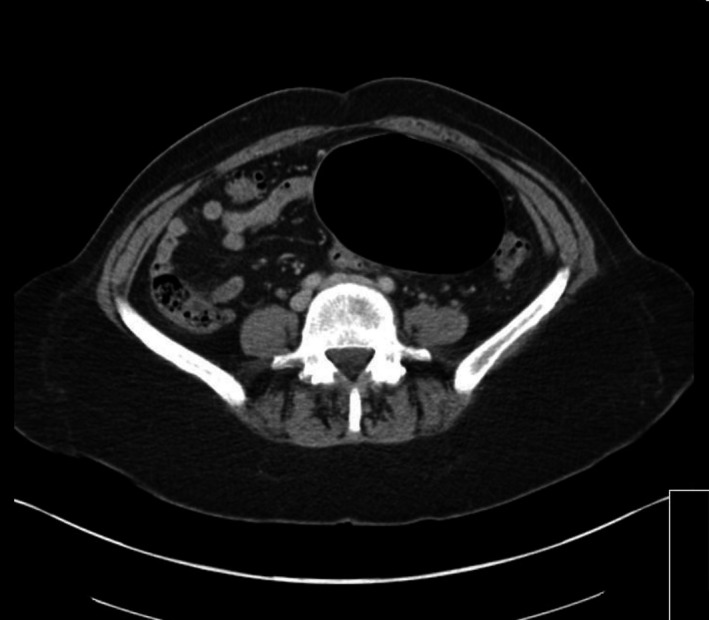
CT image (axial view).

A 58‐year‐old female referred to outpatient clinic for suspected colorectal cancer reported a 3‐week history of intermittent abdominal pain, constipation, and a self‐detected enlarging abdominal mass. She denied weight loss or rectal bleeding. However, there was a family history of bowel cancer. Clinical examination confirmed a 10 cm × 10 cm firm, nontender, suprapubic mass. Initial investigations included routine bloods, which were unremarkable, an abdominal radiograph and a CT abdomen and pelvis (Figs 1, 2, 3).

## Question

What is the diagnosis based on the imaging? How is this condition managed?

## Answer

The CT scan demonstrates a giant diverticulum arising from the sigmoid colon with extensive diverticulosis. Giant colonic diverticulum (GCD) is a rare complication of diverticulosis 1.

An acute onset occurs in a third of cases, most frequently with abdominal pain. A chronic presentation is equally common, characterized by intermittent abdominal pain, bloating, and constipation, with or without an abdominal mass. In 10% it is asymptomatic 2.

Abdominal X‐ray classically shows a large gas‐filled cyst (Balloon sign), with or without an air‐fluid level, and regular smooth walls. CT scan almost invariably provides a diagnosis, typically demonstrating a largely gas‐filled structure arising from the colon 2.

Surgical intervention, with colonic resection and *en bloc* resection of the diverticulum, is the most common and favored treatment 2. Simple diverticulectomy is not recommended due to the higher risk of dehiscence from the colonic closure 3. Conservative treatment is rarely used.

**Figure 4:**
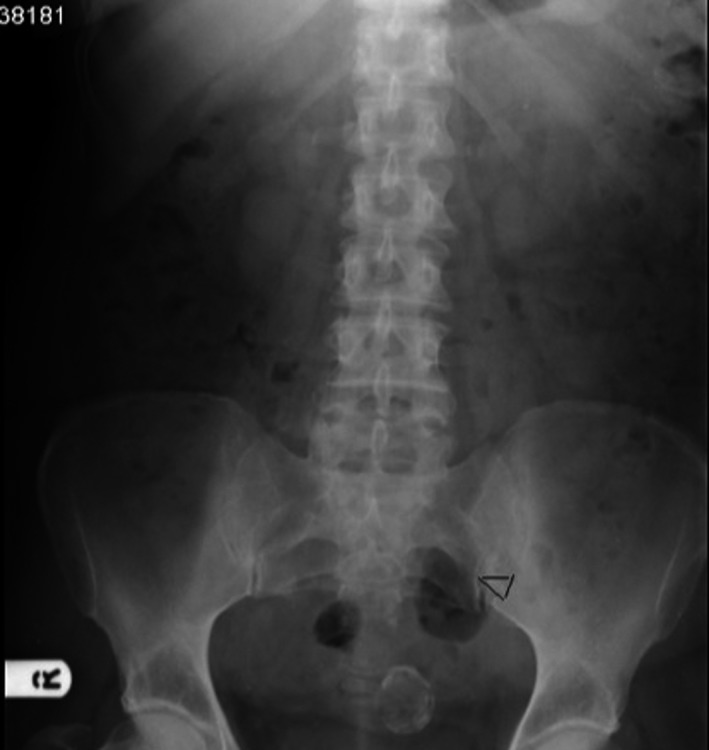
Follow‐up abdominal radiograph.

This patient requested conservative management. Four months later, she reported complete symptom resolution. The mass was no longer palpable, and there was a noticeable improvement radiographically (Fig. 4). The patient was followed up for 5 years with no return of symptoms. Although conservative treatment is an infrequent choice, it remains an option that could be considered in asymptomatic patients.

## Authorship

JP: Principal author of paper. ZP: Reviewed the paper.
